# Incidence of hereditary amyloidosis and autoinflammatory diseases in Sweden: endemic and imported diseases

**DOI:** 10.1186/1471-2350-14-88

**Published:** 2013-09-03

**Authors:** Kari Hemminki, Xinjun Li, Asta Försti, Jan Sundquist, Kristina Sundquist

**Affiliations:** 1 Division of Molecular Genetic Epidemiology, German Cancer Research Centre (DKFZ), 69120 Heidelberg, Germany; 2 Center for Primary Health Care Research, Lund University/Region Skåne, Malmö, Sweden; 3 Stanford Prevention Research Center, Stanford University School of Medicine, Stanford, CA, USA

**Keywords:** Hospitalization, Heritable amyloidosis, Periodic fever syndrome, Mutation

## Abstract

**Background:**

Amyloidoses are a heterogeneous group of progressive diseases caused by tissue deposition of misfolded proteins. According to the International Classification of Diseases, hereditary amyloidosis is divided into neuropathic and non-neuropathic forms. In Sweden, neuropathic heredofamilial amyloidosis has been identified as familial amyloidotic polyneuropathy (FAP), a fatal disease that is treated by liver transplantation. The non-neuropathic form includes familial autoinflammatory diseases. As no incidence data on these hereditary diseases are available and as even diagnostic data on non-neuropathic forms are lacking we determined the incidence of these diseases and characterized non-neuropathic conditions.

**Methods:**

Patients were identified using data from the Swedish Hospital Discharge Register and from the Outpatient Register for 2001 through 2008. All patients discharged with hereditary amyloidosis diagnoses were included and standardized incidence rates were calculated.

**Results:**

Non-neuropathic disease was diagnosed in 210 patients, with an incidence of 2.83 per million. FAP was diagnosed in 221 patients, with an incidence of 2.02 per million. Two northern provinces that are home to 5% of the Swedish population accounted for 77% of FAP cases; the incidence in one of them, West Bothnia, was 100 times that in the rest of Sweden. Approximately 98% of non-neuropathic disease patients were immigrants, most of whom were from the Eastern Mediterranean area. Young Syrian descendants had the highest incidence rate, which was over 500-fold higher than that in individuals with Swedish parents. Even the early onset of these conditions identified them as familial autoinflammatory diseases.

**Conclusions:**

FAP cases were highly concentrated in the two northernmost provinces. Non-neuropathic familial autoinflammatory diseases were of early-onset and immigrant origin most likely related to periodic fever syndromes. Paradoxically, FAP has remained endemic, in spite of population movements within the country, while familial autoinflammatory diseases, with an incidence exceeding that of FAP, were brought into the country as a result of immigration mainly from the Eastern Mediterranean area.

## Background

Amyloidosis is a heterogeneous group of diseases characterized by fibrillar protein deposits called amyloids in a single organ or multiple organs [1]. The disease nomenclature is based on the precursors of the amyloid fibrils for which at least 28 different proteins have been identified [1,2]. Amyloidosis presents in sporadic and familial forms, but incidence or prevalence data are largely lacking for all forms of the disease [3,4]. The most common and widespread form of hereditary amyloidosis is familial amyloidotic polyneuropathy (FAP), which is caused by transthyretin mutations leading to amyloid accumulation in critical organs and tissues, such as peripheral nerves, the digestive tract, the heart, and the kidneys [5,6]. The prevalence of this dominantly inherited amyloidosis is highest in Portugal, Sweden and Japan. Case numbers show large regional differences and the penetrance varies by mutation type and geography [7]. FAP is a progressive fatal disease that can be alleviated through liver transplantation, as the liver is the site of transthyretin synthesis [8]. FAP liver transplantations are carried out on a worldwide basis, and are coordinated by The Familial Amyloidotic Polyneuropathy World Transplant Registry (http://www.fapwtr.org) [9]. As of the end of 2010, 1,900 FAP transplantations had been performed. Other dominant hereditary forms of amyloidosis include apolipoprotein AI- and II-, gelsolin-, cystatin C-, lysozyme-, and fibrinogen A-related diseases, all of which are extremely rare and have relatively early onset and distinct geographical locations [5,6]. Fibrinogen A is reported to be the most common type of hereditary renal amyloidosis [10]. There are no data on hereditary amyloidosis other than FAP in Sweden, except that an expert review stated that gelsolin and apolipoprotein-related familial patients have been identified [11].

Reactive amyloidosis is a systemic form of the disease in which serum acute phase protein, amyloid A is the amyloid precursor. Reactive amyloidosis occurs as a secondary condition in patients with chronic inflammation or cancer. Many rheumatoid arthritis patients are diagnosed with reactive amyloidosis during the course of their disease [12]. Periodic fever syndromes and other inflammasome-related autoinflammatory conditions may also cause reactive amyloidosis [13-16]. Even though reactive amyloidosis may not be familial, it is a clinical manifestation of hereditary periodic fever syndromes which include pyrin-associated familial Mediterranean fever (FMF), cryopyrin-associated periodic syndrome, mevalonate kinase deficiency, and tumor necrosis factor receptor-associated periodic syndrome [13]. FMF is common in the Eastern Mediterranean area but rare elsewhere, as are the other periodic fever syndromes. A European registry on autoinflammatory diseases included 1049 patients with monogenic diseases but it reported only one patient in Sweden [17]. These diseases are normally diagnosed before age 20 years; the kidneys and the gastrointestinal tract are the vulnerable organs when the patients have developed amyloidosis [2,7,13]. According to the 10th revision of the International Classification of Diseases, hereditary autoinflammatory diseases are classified together with hereditary renal amyloidosis as ‘non-neuropathic heredofamilial amyloidosis‘, code E85.0. However, the development of amyloidosis in hereditary autoinflammatory disease patients depends on the severity of the condition and may be rare in patients responding to treatment, which for FMF includes colchicine [6,18,19]. Thus code E85.0 does not distinguish between patients with or without amyloidosis.

In this paper we addressed the lack of incidence data on these hereditary diseases using nationwide hospital discharge and outpatient data from Sweden for 2001 through 2008. The results showed that the patient characteristics for ‘non-neuropathic heredofamilial amyloidosis‘, code E85.0 showed identity with hereditary autoinflammatory diseases and we use this disease designation in the following text. There was no evidence for patients with hereditary renal amyloidosis which would also be covered by code E.85.0. In agreement, a Swedish review of 1000 consecutive kidney transplant patients revealed 189 ‘clear’ hereditary renal disorders but none were hereditary renal amyloidosis; the two amyloidosis patients belonged to a FAP pedigree from Northern Sweden [20]. The data also suggested that ‘neuropathic heredofamilial amyloidosis‘ patients (code E85.1) suffered from FAP and we use this term in the text. The results were unexpected, FAP remains a local endemic disease and hereditary autoinflammatory diseases were imported by immigrants, which reiterates the importance of international health. Some 15% of the Swedish population is born outside Sweden [21].

## Methods

### Data sources

Amyloidosis patients were identified using the Swedish Hospital Discharge Register (2001–2008) and the Outpatient Register (2001–2008). The Hospital Discharge Register has had nationwide coverage since 1987 and regional coverage since 1964. In order to ensure that the reported incidence data only included newly diagnosed cases, anyone who had been diagnosed with amyloidosis prior to 2001 was excluded. Information from the registers was linked at the individual level via the national 10-digit civic registration number assigned to each resident in Sweden for his or her lifetime. In the linked dataset, civic registration numbers were replaced with serial numbers to ensure the anonymity of all individuals.

The 10th revision of the International Classification of Diseases was used to identify disease cases using the following diagnostic codes: E85.0 for hereditary autoinflammatory disease, E85.1 for FAP and E85.2 for unspecified heredofamilial amyloidosis. Although amyloidosis diagnosis should be based on the demonstration of amyloid fibrils in tissue biopsies, it is likely that for diagnostic group E85.0, the periodic fever symptoms rather than demonstration of amyloidosis fibrils were the bases of diagnoses. The reason is the ambiguous assignment of hereditary autoinflammatory disease into this amyloidosis code. On the other hand, FAP should be correctly diagnosed and the types of transthyretin mutations are largely known in the pedigrees [7].

### Individual-level variables

Sex: Males or females. Geographic region of residence was divided into: (1) large cities (cities with a population of more than 200,000, i.e., Stockholm, Gothenburg, and Malmö); (2) Southern Sweden; and (3) Northern Sweden. Sweden is divided into 25 provinces. The border between Northern and Southern Sweden has traditionally been drawn at *Dalälven* (the Dal River, north of Stockholm), which we used to define the geographic boundary between Southern and Northern Sweden. The dataset included information on people from 64 countries and regions of birth according to the available classification.

### Statistical analysis

Person-years were calculated from the start of follow-up on 1 January 2001 until diagnosis of the relevant disease, death, emigration, or the end of the study (31 December 2008). All hereditary diseases, newly diagnosed during the follow-up period, were considered, irrespective of their possible kinship. In order to guarantee that the patients were newly diagnosed, anyone hospitalized prior to 2001 with a related diagnosis were excluded. Age-, gender-, region of residence-, immigration status-, and diagnosis subtype-specific incidence rates were calculated for the whole follow-up period. Relative weights used to calculate incidence rates were based on the European Standard Population for 2000. We used SAS version 9.2 for the statistical analyses.

### Ethical considerations

This study was approved by the Ethics Committee of Lund University, Sweden. All data were anonymous and no informed consents could be obtained.

## Results

Numbers of cases and incidence rates for the hereditary diseases are shown in Table 1 for period 2001 to 2008. Hereditary autoinflammatory disease was diagnosed in 210 patients, with a standardized incidence rate of 2.83 per million. FAP was diagnosed in 221 patients with an incidence rate of 2.02 per million after standardization. The rate was 1.8-fold higher in males than in females. Unspecified heredofamilial amyloidosis was rare; only 30 patients were diagnosed with this condition during the study period (standardized incidence rate 0.26 per million). In period 2001–2008, 68% of the patients with hereditary autoinflammatory disease were treated two or more times with the same diagnosis; for FAP the percentage was 73 and for unspecified heredofamilial amyloidosis it was 63. We wanted to characterize better unspecified heredofamilial amyloidosis by finding out if patients with this diagnosis were subsequently discharged with another related diagnosis. Among the 30 patients in Table 1, one was subsequently discharged with diagnosis FAP. Among patients diagnosed between 1997 and 2001 and not included in Table 1, 27 patients were diagnosed with unspecified heredofamilial amyloidosis and 10 of them were later diagnosed with FAP.

**Table 1:** Case numbers and incidence rates (per million person years) for hereditary diseases in Sweden, 2001-2008

	**Men**				**Women**				**All**			
**Subtype (ICD-10 code)**	**No.**	**IR**	** 95% CI**		**No.**	**IR**	** 95% CI**		**No.**	**IR**	** 95% CI**	
Hereditary autoinflammatory disease (E85.0)	116	3.11	2.54	3.68	94	2.54	2.03	3.06	210	2.83	2.45	3.22
Familial amyloidotic polyneuropathy (FAP, E85.1)	138	2.35	1.95	2.74	83	1.70	1.33	2.07	221	2.02	1.76	2.29
Heredofamilial amyloidosis, unspecified (E85.2)	15	0.25	0.13	0.38	15	0.27	0.13	0.40	30	0.26	0.17	0.35

**Figure 1:**
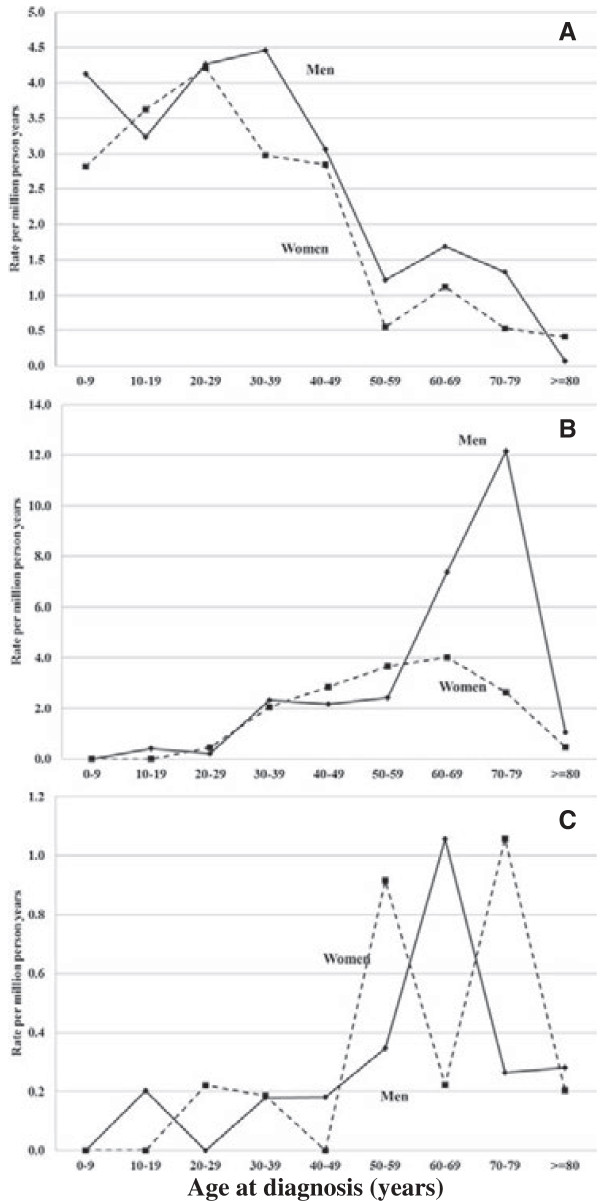
**Age-specific incidence rates (per million person-years) for amyloidosis by subtype (1997-2008). (A)** Hereditary autoinflammatory disease (E85.0). **(B)** Familial amyloidotic polyneuropathy (FAP, E85.1). **(C)** Heredofamilial amyloidosis, unspecified (E85.2).

Age-specific incidence rates for the diseases are shown in Figure 1. The incidence of hereditary autoinflammatory disease peaked at age 30 to 39 years (median age at diagnosis 32 years) in males and 20 to 29 years (median age at diagnosis 29 years) in females Figure 1A. The incidence of FAP peaked sharply in men at age 70 to 79 years (median age at diagnosis 67 years) and less sharply in women 10 years earlier (median age at diagnosis 57 years) (Figure 1B). Unspecific heredofamilial amyloidosis was also found to be a disease of old age; the median age at diagnosis was 66 years for men and 64 years for women (Figure 1C).

Differences in the incidence of these diseases according to region of residence in 1990 are shown in Table 2. Data for regions where the incidence was more than twice the overall incidence in Sweden are shown. For hereditary autoinflammatory disease, none of the 25 provinces showed an increase because close to half of patients resided in an ‘unspecified residential area’. The only significant differences in incidence rates compared with the overall incidence in Sweden for FAP were for *Västerbotten* (West Bothnia) and *Norrbotten* (North Bothnia); for unspecified heredofamilial amyloidosis, *Västerbotten* showed a non-significant difference from the overall incidence in Sweden. These two provinces are located in the most northern part of Sweden. North Bothnia is the northernmost province and West Bothnia borders it to the south. Remarkably, the two provinces together accounted for 77% of FAP cases in Sweden. The incidence of FAP in West Bothnia was 22 times higher than the national average. Moreover, as the incidence outside West Bothnia and North Bothnia was only 0.45 per million (N = 50, 95% CI 0.33-0.57); the incidence in West Bothnia of 45.28 per million was 100 times higher than that in the rest of the country.

**Table 2:** Case numbers and incidence rates (per million person years) for the hereditary diseases by region of residence

	**Hereditary autoinflammatory disease (E85.0)**				**Familial amyloidotic polyneuropathy (FAP, E85.1)**				**Heredofamilial amyloidosis, unspecified (E85.2)**			
	**No.**	**IR**	** 95% CI**		**No.**	**IR**	** 95% CI**		**No.**	**IR**	** 95% CI**	
Jönköping									4	0.92	0.02	1.81
Västerbotten (West Bothnia)					122	45.28	37.25	53.32	4	1.29	0.03	2.55
Norrbotten (North Bothnia)					49	13.16	9.48	16.85				
Unspecified	102	7.96	6.42	9.51								
All	210	2.83	2.45	3.22	221	2.02	1.76	2.29	30	0.26	0.17	0.35

A large proportion of patients with hereditary autoinflammatory disease had unspecified residential area in 1990, and we suspected that the reason was that some of them were immigrants. Table 3 shows the country of birth for patients diagnosed with hereditary autoinflammatory disease. More than half of the patients were foreign-born, with immigrants from Turkey, Lebanon and Syria constituting the largest groups (each contributing 20 or more patients). Syrians had the highest incidence rate (109.40 per million). All the listed nationalities had significantly higher incidence rates compared to Swedes. A closer look at the 95 Swedish-born individuals showed that both parents of only 9 of them were born in Sweden; however, the ethnicity of these parents could not be confirmed. The incidence of hereditary autoinflammatory disease in Swedish-born individuals whose parents were both born in Sweden was 0.25 per million. Among the second-generation immigrants with compatriot parents, those of Syrian descendent showed the highest incidence (136 per million, i.e., over 500 times higher than the incidence in individuals with two Swedish-born parents). There were too few cases to draw conclusions about differences in incidence rates between first- and second-generation immigrants.

**Table 3:** Case number and incidence rates (per million person years) for hereditary autoinflammatory disease by birth country of the first generation

	**First generation**				**Second generation of compatriot parents**			
**Birth country**	**No.**	**IR**	** 95% CI**		**No.**	**IR**	** 95% CI**	
Sweden	95	1.48	1.18	1.78	9	0.25	0.09	0.41
Turkey	20	50.17	28.18	72.16	14	38.10	18.14	58.06
Libanon	22	74.70	43.48	105.92	11	99.40	40.66	158.14
Iran	12	35.40	15.37	55.43	5	12.60	1.56	23.64
Syria	20	109.40	61.45	157.35	15	136.40	67.37	205.43
The United Arab Emirates	5	59.90	7.40	112.40	1	23.97	0.00	70.95
Armenia/Azerbaijan/Georgia*	5	44.90	5.54	84.26	0			
All	210	2.83	2.45	3.22				

Countries included when at least 3 patients in the first generation had immigrated.

*Additional countries: Kazachstan/Kirgizistan/Tadzjikistan/Turkmenistan/Ukraine/Uzbekistan/Belarus.

**Figure 2:**
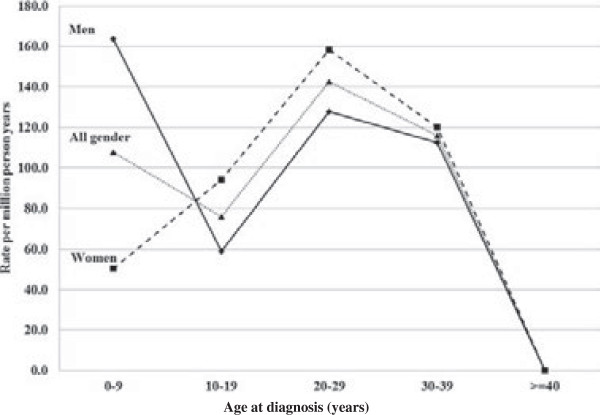
Age-specific incidence rates (per million person-years) for hereditary autoinflammatory disease (E85.0) among second-generation immigrants with compatriot parents from Turkey, Lebanon, and Syria (based on 22 males, 18 females).

Age-specific incidence rates for second generation immigrants of Turkish, Lebanese and Syrian descent are shown in Figure 2. The incidence for both genders combined peaked at age 20 to 29 years. The median age at diagnosis for both genders combined was 14 years (11 years for males, 19 years for females).

## Discussion

The advantages of the present study include nationwide coverage in a country with universal access to medical services of high diagnostic standards. Amyloidosis diagnostics are time-consuming and it has been reported that a small proportion of hereditary amyloidosis is misdiagnosed as the common immunoglobulin light chain amyloidosis [22]. However, FAP is well known in Sweden and, moreover, two thirds of the patients were discharged more than once with the same specific amyloidosis subtype which implies consistent diagnostics. Also, the striking finding showing the precise geographic location of FAP cases and the immigrant dominance of hereditary autoinflammatory disease testify to a high diagnostic accuracy. Based on the similar geographic location, approximately similar age distribution and interchanged diagnosis in many patients of FAP and unspecified heredofamilial amyloidosis, they may be the same disease, with a joint incidence of 2.28 per million, somewhat less than that of hereditary autoinflammatory disease (2.83 per million). These incidence rates refer to inpatients and outpatients for years 2001 to 2008.

Swedish neuropathic heredofamilial amyloidosis (FAP) is caused by any of three identified transthyretin mutations. However, even for the same mutation penetrance and manifestations vary [7]. The mean age of onset has been reported to be 56 years, which is somewhat lower than what we found here (the median age at diagnosis was 67 years for men and 57 years for women). The likely explanation is that symptoms do not immediately lead to hospitalization, but once patients are hospitalized amyloidosis is fulminant. However, considering the variable penetrance of FAP, the present incidence rates underestimate the incidence of FAP. Our data showed that 77% of the Swedish FAP patients were diagnosed in the two northernmost provinces of Sweden (West and North Bothnia), which together account for slightly more than 5% of the Swedish population. The incidence of FAP was over 100-fold higher in West Bothnia than in the rest of the country (excluding North Bothnia), in spite of large population movements within Sweden since the Second World War.

Autoinflammatory diseases are caused by dysfunction of the inflammasome complex of the innate immune system [13,14]. They include several monogenic diseases such as pyrin-associated FMF, cryopyrin-associated periodic syndrome, mevalonate kinase deficiency, and tumor necrosis factor receptor-associated periodic syndrome. Many of these diseases are diagnosed by pediatricians and, for example, 80% of FMF cases occur before age 20 years [13]. FMF is, according to a review, almost always restricted to Turks, Armenians, Arabs and non-Ashkenazi Jews or emigrants with these ethnicities and, according to this source, no cases have been described in individuals of Scandinavian origin [18]. Autoinflammatory conditions may give rise to reactive amyloidosis, which may manifest initially as renal problems; the amyloid precursor is serum amyloid A, the normal and not a mutated protein [13-16,18]. Our first clue that code E85.0 included periodic fever syndromes was the overwhelming proportion of patients who were immigrants from the Eastern Mediterranean area, with the incidence being highest in first-generation Syrian immigrants (109 per million) and their descendants (136 per million). In endemic areas, rates have been estimated to be as high as 1,000 per million [18]. The relatively early age of onset also supported the disease identity of periodic fever syndrome; the highest incidence rates were in the 20 to 29 years age group in second-generation immigrants (median age at diagnosis 14 years). However, it is important to note that the immigrant populations originating from the Eastern Mediterranean area are relative newcomers to Sweden and their oldest descendants are still young adults [23]. Thus, the calculated median ages at hospitalization are likely to be too high in the first generation immigrants who entered Sweden at around age 25 years and who could have the disease already at immigration. The delay between first disease episodes and diagnosis was over 7 years in the Eurofever registry [17]. The calculated median ages may be too low for the young second generation because of the age truncation (still a young population).

An obvious question resulting from the above findings is whether native Swedes have indigenous hereditary autoinflammatory or related diseases. Among the 210 identified patients, 95 were born in Sweden but for only nine (2.3%) had both parents been born in Sweden. However, we were unable to confirm parental ethnicity. Recent data demonstrated that some inflammasome-related conditions appear to have indigenous origins in Sweden. Cryopyrin-associated periodic syndrome cases diagnosed in adulthood have been reported and common inflammasome-related *NLRP3* and *CARD8* polymorphisms have been described, although without evidence on disease susceptibility [15,16]. Cases of pediatric periodic fever, aphthous stomatitis, pharyngitis and adenitis (PFAPA) have also been described in Sweden, but the disease is apparently not heritable; mevalonate kinase deficiency has not been diagnosed in ethnic Swedes [24]. Taken together, the available data suggest that hereditary autoinflammatory disease, as described in the present study, is a disease of immigrants originating from the Eastern Mediterranean area.

## Conclusions

In conclusion, this nationwide hospital in- and outpatient study showed an incidence of 2.02 per million for FAP and 2.83 per million for hereditary autoinflammatory disease. Both of these diseases showed distinct characteristics. FAP cases were concentrated in the two northernmost provinces of Sweden, suggesting that this disease entity only constitutes FAP with transthyretin mutations and practically excluding other types of diseases. Hereditary autoinflammatory disease was found to be an early-onset disease of immigrant origin. However, the identities of the type(s) of periodic fevers could not be established. Paradoxically, FAP has remained endemic, in spite of population movements, while hereditary autoinflammatory disease has been brought into the country as a result of population movements. We have recently shown that Swedish FAP patients have an increased risk of non-Hodgkin lymphoma (Hemminki et al. unpublished observation).

## Abbreviations

FAP: Familial amyloidotic polyneuropathy; IR: Incidence rate; CI: Confidence interval.

## Competing interests

The authors declare that they have no competing interests.

## Authors’ contributions

KH, XJ and KS designed the study, JS, KS, XJ and KH gathered and analyzed the data, KH, AF and XJ wrote the report and all authors comments on the manuscript. KH confirms that he had full access to all the data in the study and had final responsibility for the decision to submit for publication. All authors agreed to publishing the paper. All authors read and approved the final manuscript.

## Pre-publication history

The pre-publication history for this paper can be accessed here:

http://www.biomedcentral.com/1471-2350/14/88/prepub
